# Preliminary Test for 3D Surface Strain Measurement in the Tower and Foundation of Offshore Wind Turbines Using DOFS

**DOI:** 10.3390/s23156734

**Published:** 2023-07-27

**Authors:** Taolue Yang, Tao Tao, Xinran Guo, Yi Yang, Shi Liu

**Affiliations:** China Southern Power Grid Technology Co., Ltd., Guangzhou 510080, China; yang_taolue@163.com (T.Y.); g18820101803@163.com (X.G.); yyxt007@sina.cn (Y.Y.)

**Keywords:** OFDR, optical fiber sensor, strain/temperature field reconstruction, structural health monitoring, wind turbines, experimental validation

## Abstract

Subjected to the relentless impacts of typhoons and rough seas, offshore wind turbines’ structures, particularly the tower, foundation, and blade, are at constant risk of damage. Full-field strain monitoring helps to discover potential structural defects, thereby reducing disasters caused by overall structural failure. This study introduces a novel method for assessing strain and temperature fields on these kinds of 3D surfaces of cylindrical structures. The method harnesses the capabilities of a high spatial resolution (0.65 mm) Optical Frequency Domain Reflectometer (OFDR)-based Distributed Optical Fiber Sensor (DOFS) in conjunction with a unique helical wiring layout. The core process begins with mapping the fiber optic path onto a plane corresponding to the unfolded cylinder. Fiber optic signals are then differentiated on this plane, deriving a two-dimensional strain distribution. The plane strain field is subsequently projected onto the 3D side of the cylinder. An experiment was carried out in which a 3.5 m long optical fiber was helically wound with a 10 mm pitch on the surface of a cantilever beam of a cylinder shell with a diameter of 36 mm and a length of 300 mm. The experiment collected about 5400 measurement points on the cylindrical surface of 340 cm^2^, approximately 15.9 measurement points per square centimeter. The reconstructed results successfully reveal the strain field of the pipe cantilever beam under bending and torsional loads, as well as the palm-shaped temperature field. This experimental validation of the method’s efficacy lays the theoretical groundwork for its application to real wind turbines.

## 1. Introduction

Offshore wind turbines are persistently exposed to adverse conditions such as salt mist, high temperature, high humidity, and lightning, leading to surface corrosion and peeling or internal stress concentration in structural components, thereby reducing their strength and lifespan. These turbines are continuously subjected to loads from strong winds, ocean waves, and sea currents, which can cause fatigue cracking or buckling instability in the structural components [1]. Under extreme conditions such as typhoons and tsunamis, offshore wind turbines might experience enormous impact forces or displacement deformations beyond their design range, triggering holistic damage or failure to the main structure, such as blade fracture, mooring system failure, foundation tilting, or tower buckling. Consequently, monitoring the in-service damage of offshore wind turbine structures is crucial for ensuring their safe operation [2].

For the structural health monitoring of wind turbine units, a variety of methods are available, including acoustics, thermography, ultrasonics, vibration modalities, and stress-strain monitoring. However, each of these techniques possesses certain limitations when applied in marine environments, thereby restricting their usage. The acoustic emission [3] method uses the elastic energy released during the structural failure process to infer the location and extent of the damage. However, due to noise interference, its effectiveness in damage characterization and assessment is not optimal. Based on the principle that thermal conductivity anomalies occur at material defect sites, the thermographic method [4] utilizes infrared cameras to measure temperature distribution for defect detection. However, in marine environments, the impacts of airflow and thermal radiation on the temperature field make this method difficult to apply. The ultrasonic method [5] sends ultrasonic waves into the material and analyzes the signals reflected by defects or cracks to determine their location and size. However, it can only be used when the turbine is not in operation, and thus it does not enable real-time monitoring. Furthermore, the large-scale structure monitoring presents high difficulty and cost. Taking advantage of the fact that structural damage leads to changes in modal parameters due to reduced mass or stiffness, the vibration modal method [6] identifies modal changes through multiple accelerometer sensors and spectral analysis. Most of these studies are conducted under laboratory conditions and are challenging to implement on full-size turbines and in the presence of external interference [7].

**Figure 1:**
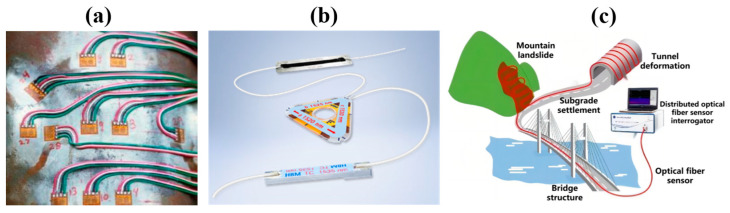
Three types of structural strain monitoring sensors: (**a**) multi-point measurement with resistive strain gauges involves a large number of wires; (**b**) multiple Fiber Bragg Gratings (FBGs) can be cascaded on a single optical fiber; (**c**) distributed fiber optic sensors can measure over tens of kilometers with a single optical fiber.

Strain-based structural health monitoring methods have been widely validated and applied in various engineering fields. The strain monitoring method [8] detects damage by identifying abnormalities in local or overall stress states caused by defects. Commonly used strain gauges can only be placed near the areas anticipated to undergo significant strain [9]. However, defect locations in large structures like wind turbines are often hard to predict. Moreover, deploying a large number of strain gauges with required test cables can alter the dynamic characteristics of the structure, as shown in Figure 1a, which is not conducive to long-term safe operation. LoRa^TM^ technology offers a wireless signal transmission method that circumvents the cost and performance implications of extensive cable deployment in wide-ranging, multi-point monitoring scenarios [10,11]. However, it is not without its limitations. Primarily, it has a low data transmission rate, falling short in scenarios requiring high-speed data transfer. Furthermore, despite LoRa’s relatively low power consumption, battery life and energy consumption still become issues during prolonged, continuous data transmission.

The aforementioned electrical sensing probes, including acoustic probes, thermal imagers, accelerometers, and resistive strain gauges, face many difficulties in corrosive marine environments. For instance, seawater contains a large amount of salts and microorganisms, which can accelerate the corrosion of metal materials and electrochemical reactions, thereby affecting the stability and reliability of the sensors and circuits. This necessitates that the sensors must have high strength, high sealing, and high corrosion resistance. They also must exhibit good mechanical stability and reliability.

The overall fatigue cracking or buckling of a structure generally originates from local minor cracks and fatigue damage within the material. These local material defects can occur anywhere in the structure, making them difficult to locate and monitor. Without intervention or repair, these defects gradually enlarge, eventually leading to comprehensive structural failure. The most notable characteristic of defect generation and growth is the changing strain patterns surrounding the defect. Effectively monitoring these strain changes is the most potent means of detecting and locating these defects. Therefore, utilizing distributed optical fiber for full-field strain monitoring offers a highly promising method for structural health monitoring.

Fiber optic sensors possess many advantages over traditional electrical sensors, such as compactness, lightweight, strong interference resistance, and high reliability in marine corrosive environments [12,13,14]. Moreover, they can achieve distributed or multi-point simultaneous measurements, as shown in Figure 1b,c. Structural damage monitoring methods based on fiber optic strain sensing technology have been widely applied in civil engineering, aerospace, power grids, and oil and gas extraction [15,16,17,18,19]. In recent years, they have also been extensively used in the temperature and strain monitoring of offshore wind turbines.

Thanks to the important breakthroughs made by the LUNA company in the commercialization of the Optical Frequency Domain Reflectometer (OFDR) [20,21] in the past ten years, researchers have gradually realized its advantages in wind turbine structural health monitoring. The Distributed Optical Fiber Sensor (DOFS) based on OFDR can achieve: a large range; continuous, high spatial resolution; high accuracy; and real-time, long-term strain/temperature network array monitoring on large structures, showing enormous application potential in wind turbines structure monitoring; and some preliminary research work has also received widespread attention. Shajiee et al. [22] have attempted to use OFDR distributed fiber optic temperature sensors for monitoring blade icing and combine it with a PID heating device for de-icing. Zhang et al. [23] have successfully used optical fiber for full-length mechanical load monitoring of wind power pile foundations. Lally et al. [24] have carried out fiber optic testing research on the deformation of wind turbine blade structures.

Strain field measurement methods based on DOFS have also been studied to a certain extent. Grave et al. [25] demonstrated how standard optical fibers, when bonded to a surface or embedded in a laminate, can measure strain fields along their entire length, using the optical backscatter reflectometer. The DOFS was successfully embedded and bonded to a composite joint, enabling the identification of adhesive damage. Choi and Kwon [26] expanded on this method to map impact damage through the strain distributed in an optical fiber embedded in a composite cylinder. This approach yielded accurate mapping images for identifying impact locations and severities in composite cylinders. Sierra-Pérez et al. [27] introduced a damage detection method for wind turbine blades based on strain field pattern recognition. The researchers embedded optical fiber sensors in blade structures, allowing for the detection of defects and nonlinearities and thus preventing premature structural failure. Tan et al. [28] proposed another method using DOFS for measuring strain distributions to detect and reconstruct buckling deformation in thin-walled plates, demonstrating its practicality and effectiveness. Hubbard et al. [29] used distributed acoustic sensing (DAS) to monitor the vibration characteristics of wind turbine towers, but they highlighted that the spatial resolution of current DAS systems is poor and significantly affected by noise interference, impeding precise detection of structural defects. In addition, preliminary results have been achieved in basic research on OFDR fiber calibration in extreme environments [30], strain transfer analysis [31], force-heat decoupled measurement [32], and wiring configuration [33].

At present, achieving effective full-field strain monitoring on offshore wind turbines remains a considerable engineering challenge due to issues such as electromagnetic interference and corrosion, commonly experienced with traditional electrical sensors in marine environments. While FBG can counter these issues, its single-point measurement cannot adequately encompass the entire turbine structure. Although some research has explored DOFS for wind turbine monitoring, these have yet to deliver the desired practical results due to some unresolved technical problems.

Our study explores the application of OFDR-based DOFS to address this challenge, offering a promising solution by enabling strain field measurements on the surfaces of cylindrical structures. We mathematically derived a planar projection relationship under spiral wiring and performed differential calculations on fiber signals to determine the strain field on cylindrical surfaces. Our laboratory experiments used a small cylindrical shell sample to validate the effectiveness of our strain reconstruction process, which included fiber pasting, signal collection, data processing, and visualization methods. This innovative strain monitoring approach provides a robust theoretical and technical foundation for future strain measurement experiments in real-world wind turbine towers and foundations, marking a significant step forward in offshore wind turbine monitoring.

## 2. The Temperature/Strain Field Reconstruction Method

There are many types of optical fiber sensing, most of which are still in laboratory research, and a few have begun to be applied in engineering practice. At present, in the strain/temperature monitoring scenario, the most potential application is the Fiber Bragg Grating (FBG) and OFDR. FBG can only achieve single-point monitoring, while OFDR can achieve continuous monitoring with high spatial resolution, enabling more complex measurement modes. Both are similar in sensitive principle [34].

### 2.1. Optical Fiber Sensing Principle

**Figure 2:**
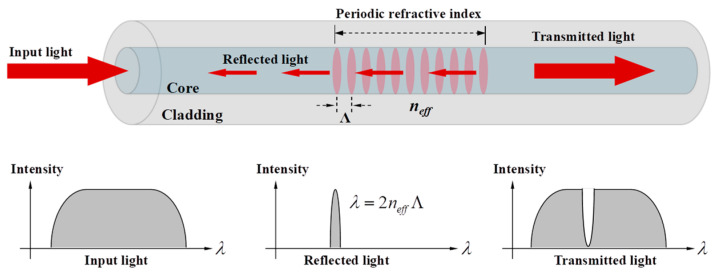
Principle of Fiber Bragg Grating (FBG) Sensing.

In FBG, as shown in Figure 2, the periodic (Λ) change in refractive index ($n_{eff}$) along the fiber core length will strongly reflect light of a specific wavelength (λ). This wavelength is known as the central wavelength of the FBG and is related to temperature/strain. Demodulating the central wavelength can be used for sensing. Writing multiple FBGs with different central wavelengths at different locations on the same fiber can realize discrete quasi-distributed sensing, a technique known as wavelength division multiplexing. However, the reflection spectrum of FBGs is influenced by various factors, such as fiber materials, temperature, strain, etc., causing instability in the peak position and intensity of the reflection spectrum, which adds complexity and error to signal processing. Moreover, crosstalk exists among FBGs, where the reflected signal of one FBG affects the reflected signals of other FBGs, resulting in signal distortion and overlap. As the number and density of FBGs increase, the crosstalk phenomenon intensifies, limiting the maximum quantity and minimum spacing of FBGs in a single fiber. This restriction makes it challenging to achieve large-scale, high spatial resolution array-based measurements.

**Figure 3:**
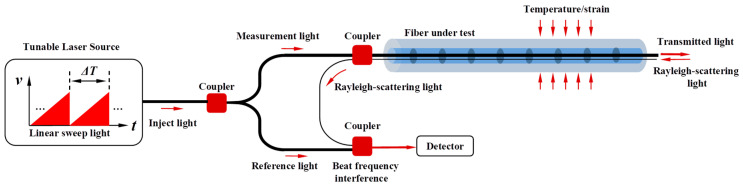
Principle of Optical Frequency Domain Reflectometry.

Unlike FBG, which can only achieve single-point measurement, OFDR can achieve continuous measurement with high spatial resolution along the length of the fiber. As shown in Figure 3, the linear sweep light generated by the tunable laser source is divided into measurement light and reference light by the coupler. When the measurement light enters the test fiber, Rayleigh scattered light backpropagating will be generated at each point, and the beat frequency interference will occur with the reference light in the coupler and be detected by the detector. The Rayleigh scattering spectrum (RBS) at each point of the fiber can be obtained through Fourier analysis. Under the action of temperature and strain, the RBS shift (which can be converted into the corresponding optical wavelength shift) follows the same principle as FBG.

By using ordinary single-mode optical fiber, OFDR can achieve millimeter-level spatial resolution temperature/strain sensing within a 100-m range, with strain measurement accuracy of ±1με, temperature measurement accuracy of ±0.2 °C, and dynamic measurement frequency of 200 Hz [20]. Due to the dense and nearly continuous measurement points and the combination of high accuracy, high sensitivity, high stability, and anti-electromagnetic interference, Rayleigh scattering OFDR fiber technology can easily handle the measurement tasks of non-uniform strain and temperature fields in complex structures, making it the most advanced fiber optic strain/ temperature measurement equipment.

The load of strain and temperature is reflected in changes in the refractive index of the fiber due to the photo-elastic effect, thermo-optic effect, and the elongation of the fiber. The OFDR system demodulates the frequency change of backscattered Rayleigh light in the fiber to obtain this refractive index change, thus realizing strain and temperature sensing. The relationship between the shift of light frequency (Δν ), the mechanical strain (Δε ) in the length direction of the fiber, and the change in temperature (ΔT ) can be represented as

(1) $$-\frac{\Delta \nu }{\nu_{0}}=K_{F}\Delta \varepsilon +K_{T}\Delta T;K_{F}=1-Pe;K_{T}=\left(1-Pe\right)\alpha_{S}+\xi$$

where $\nu_{0}=1.935\times 10^{5}\mathrm{GHz}$ represents the center frequency of the initial light wave, $K_{F}$ is the strain sensitivity coefficient, $K_{T}$ is the temperature sensitivity coefficient, $\xi =6.65\times 10^{-5}/℃$ is the thermo-optic coefficient of the glass, Pe=0.217 is the photo-elastic coefficient of the glass, and $\alpha_{S}$ is the thermal expansion coefficient of the measured structure’s material.

**Figure 4:**
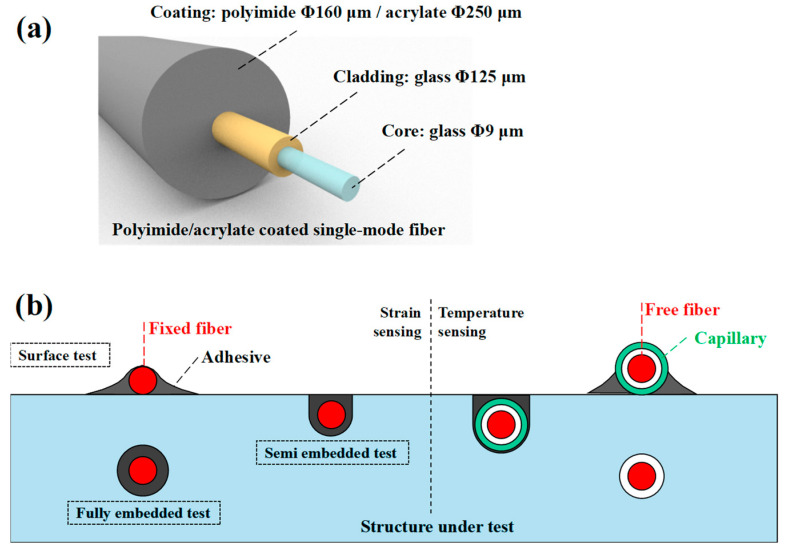
Fiber Optic Structure: (**a**) structure of Single-Mode Fiber; (**b**) installation for strain/temperature measurement.

Equation (1) reveals that the shift in light frequency is determined by the mechanical strain and changes in temperature. As depicted in Figure 4b, when measuring temperature, the free fiber is typically placed within a capillary to avoid the effects of strain, and hence the frequency shift corresponds to changes in temperature [28]. During strain measurement, the fiber is often bonded on the structure, making it challenging to avoid temperature interference. Therefore, an additional temperature-measuring fiber is typically incorporated to compensate for frequency shifts induced by temperature changes.

### 2.2. Installation of Optical Fiber Sensors

The optical fiber sensor, with a diameter of 160 or 250μm, as shown in Figure 4a, is very fragile and can be easily mechanically damaged or chemically corroded. Therefore, appropriate encapsulation structures and adhesives are needed to protect it and make it tightly coupled with the measured structure. Depending on different test environments and purposes, optical fiber sensors can adopt different installation methods such as surface-mounted, partially embedded, and fully embedded, as shown in Figure 4b. The surface-mounting method is to adhere the fiber directly to the surface of the measured structure, which is simple and convenient, but prone to external interference and pollution. The semi-embedded method is to embed the optical fiber in the small groove on the surface of the measured structure, which can improve signal stability and sensitivity. The fully embedded method is to embed the fiber completely into the inside of the measured structure, which can protect the fiber to the maximum extent and improve signal quality, but requires significant modification of the measured structure, or the fiber must be embedded at the prefabrication stage.

Choosing the right adhesive based on different testing environments is crucial. For example, in low-temperature measurement environments, ordinary adhesives that are not low-temperature-resistant are prone to cracking and de-bonding. At high temperatures, they may soften, and in marine environments, they may suffer from corrosion. All these factors can severely affect measurement accuracy and even lead to measurement failure.

In wind turbine tower tubes and pipe pile foundations, optical fiber sensors can be installed by adhering to the inner surface of the structure. This construction method is simple and easy to implement and can be quickly deployed in existing wind turbines. Another possible method is to embed the fiber optic sensors into the component matrix during the manufacturing process of the blades and tower tubes. By real-time monitoring of the stress, strain, and temperature parameters of key structural components, fiber optic sensing technology can help identify and predict potential damage in advance, thereby improving the reliability and operational efficiency of wind turbines. This monitoring method is significant in ensuring structural integrity, reducing maintenance costs, and extending service life.

**Figure 5:**
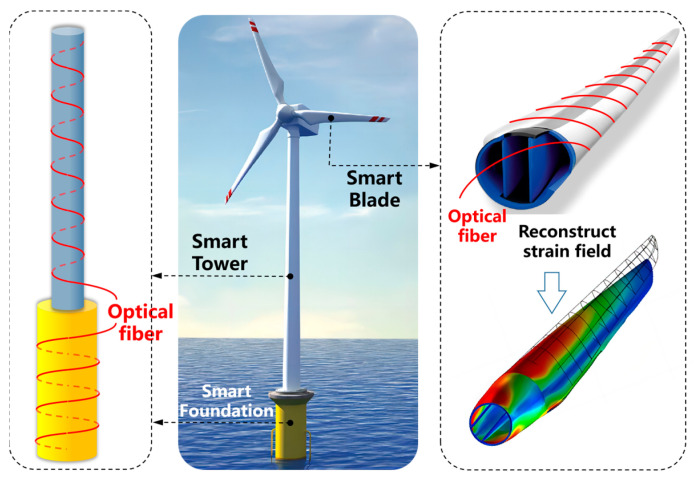
Installing distributed fiber optic sensors on the tower, foundation, and blades of an offshore wind turbine creates a smart structure with wide-range sensing capabilities.

Offshore wind power structures mainly consist of components such as foundations, tower tubes, and blades, and are mainly approximately cylindrical structures. Fibers can be densely arranged on the structural surface in a spiral winding manner, and the densely arranged measurement points on the surface can be used to reconstruct the surface strain field. The layout configurations of the blades, tower tubes, and pipe pile foundations are shown in Figure 5. By real-time collection and analysis of these measurement signals, the strain field distribution on the structural surface can be obtained, and further calculation of the axial force, shear force, bending moment, deflection, and other key parameters that the structure bears can be made. The strain information also reflects the internal stress state of the tower tube and the possible damage.

### 2.3. Reconstruction Principle of the Measuring Signal

**Figure 6:**
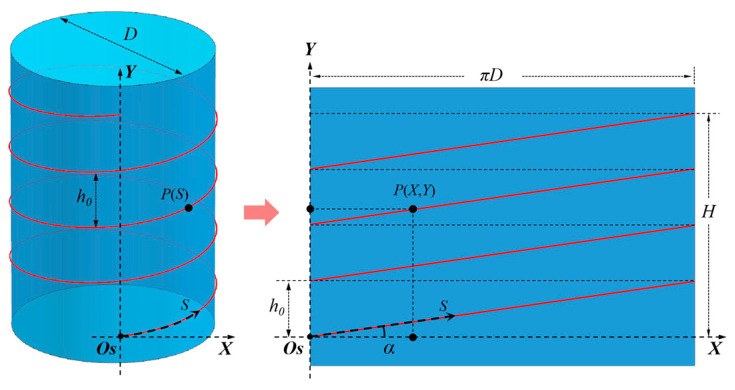
Projection of optical fiber on the side of the cylinder.

On the surface of a cylinder, a single optical fiber can cover more measurement areas by helical wiring than a straight line, but it introduces difficulties in the reconstruction of the strain field. As shown in Figure 6, the fiber is arranged in a cylindrical spiral line S, with Os as the starting point, $h_{0}$ as the pitch, and D as the diameter, ascending counterclockwise. Expand the cylindrical side to obtain a rectangular surface with the straight-line Os-Y on the side of the cylinder as the boundary, and describe the expanded rectangular surface with a two-dimensional rectangular coordinate system Os-X-Y. The spiral line on the cylindrical surface is projected onto the rectangular surface to become several separated straight lines, with X-direction length πD and Y-direction height $h_{0}$, and an inclination angle $\alpha =\arctan\left(h_{0}/\pi D\right)$.

A point P (S) on the fiber will be projected onto the rectangular surface P (X, Y), and the Y-coordinate can be expressed as

(2) $$Y_{\left(S\right)}=S\sin\alpha =\frac{S}{\sqrt{1+{\left(\frac{\pi D}{h_{0}}\right)}^{2}}}$$

To find the X-coordinate, first perform the modulo operation on S for a single pitch arc length $\sqrt{h_{0}^{2}+{\left(\pi D\right)}^{2}}$ as follows:

(3) $$S*=Mod\left[S,\;\sqrt{h_{0}^{2}+{\left(\pi D\right)}^{2}}\right]$$

The obtained X-coordinate is represented as

(4) $$X_{\left(S\right)}=S*\cos\alpha =Mod\left[S,\;\sqrt{h_{0}^{2}+{\left(\pi D\right)}^{2}}\right]/\sqrt{1+{\left(\frac{h_{0}}{\pi D}\right)}^{2}}$$

**Figure 7:**
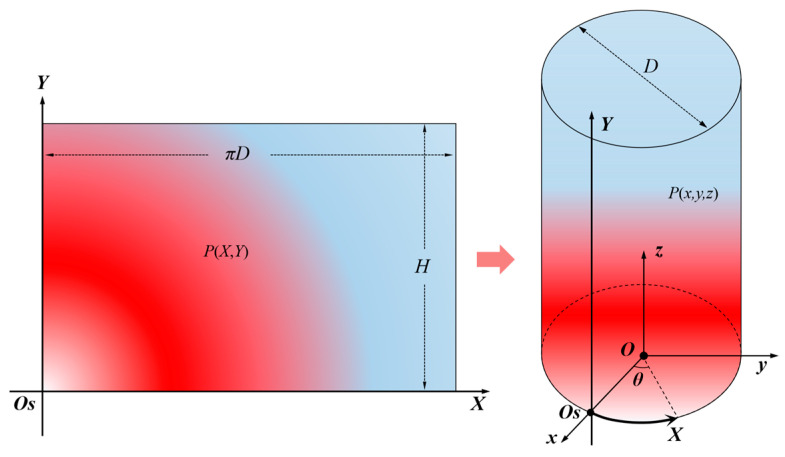
The 2D planar signal projection onto the cylindrical 3D cylinder surface.

At this point, the fiber S curve measurement signal $\left[S,F_{\left(S\right)}\right]$ has been converted into a series of parallel straight-line signals separated on a two-dimensional rectangular plane. The data format is a table represented in the form of fiber position parameter S, $\left[X_{\left(S\right)},Y_{\left(S\right)},F_{\left(S\right)}\right]$, where $F_{\left(S\right)}$ is the original fiber test signal RBS shift. Through two-dimensional interpolation, the one-dimensional signal of multiple parallel fibers $F_{\left(S\right)}$ can be extended to a rectangular two-dimensional plane signal $F_{\left(X,Y\right)}$, given in the form of a two-dimensional matrix ${Μ}_{F,\;n_{X}\times m_{Y}}$. The size $n_{X}\times m_{Y}$ of the matrix can be customized according to the data density. The row and column numbers can be converted to X and Y coordinates, respectively, and the numerical value of the corresponding position element is the fiber test signal $F_{\left(X,Y\right)}$, that is, the test data $\left[X,Y,F_{\left(X,Y\right)}\right]$ at any point on the rectangular two-dimensional surface, where 0≤X≤πD and 0≤Y≤H. Next, project the obtained rectangular plane two-dimensional test signal onto the cylindrical surface, as shown in Figure 7.

In the Cartesian coordinate system Oxyz established at the center of the cylindrical bottom surface, the equation of the cylindrical surface with rectangle sides X and Y as parameters can be represented as

(5) $$\left\{ \begin{array}{l} x_{\left(X,Y\right)}=\frac{D}{2}\cos\theta =\frac{D}{2}\cos\left(\frac{2X}{D}\right)\\ y_{\left(X,Y\right)}=\frac{D}{2}\sin\theta =\frac{D}{2}\sin\left(\frac{2X}{D}\right)\\ z_{\left(X,Y\right)}=Y \end{array}\right.$$

and it satisfies 0≤X≤πD and 0≤Y≤H.

Drawing graphics using software requires discretizing the data first. Set the parameter X step length to $\pi D/n_{X}$, and the parameter Y step length to $H/m_{Y}$, and the matrix of the cylindrical surface coordinates (x,y,z) is ${Μ}_{x,\;}{}_{n_{X}\times m_{Y}}$, ${Μ}_{y,\;}{}_{n_{X}\times m_{Y}}$, ${Μ}_{z,\;}{}_{n_{X}\times m_{Y}}$. Using Origin software as an example, a three-dimensional graph of the cylindrical surface is drawn, $\left[{Μ}_{x,\;}{}_{n_{X}\times m_{Y}},{Μ}_{y,\;}{}_{n_{X}\times m_{Y}},{Μ}_{z,\;}{}_{n_{X}\times m_{Y}}\right]$, and then the test data ${Μ}_{F,\;n_{X}\times m_{Y}}$ are projected onto the cylindrical surface according to the color.

In engineering, measurements are widely carried out in the cylindrical surface with the spiral winding method, such as in oil and gas pipelines, tunnels, circular section beams and columns, etc. First, convert the test signal from the spiral line curve coordinate system Os-S to the rectangular two-dimensional Cartesian coordinate system Os-X-Y after the side is expanded. Then perform two-dimensional data interpolation operations to obtain the test signals of the entire side. Finally, project onto the cylindrical shell in the three-dimensional Cartesian coordinate system Oxyz to complete the signal reconstruction.

(6) $$\left[S,F_{\left(S\right)}\right]\to \left[X_{\left(S\right)},Y_{\left(S\right)},F_{\left(X,Y\right)}\right]\to \left[x_{\left(X,Y\right)},y_{\left(X,Y\right)},z_{\left(X,Y\right)},F_{\left(x,y,z\right)}\right]$$

## 3. Test Bench

In order to verify the effectiveness of the proposed reconstruction method, we designed and conducted a series of experiments aimed at testing the temperature/strain field measurements and reconstruction of cantilever hollow cylindrical beams under bending, torsion, bending-torsion combination, and temperature loads. These experiments will help validate the effectiveness of the proposed method in offshore wind turbine testing.

**Figure 8:**
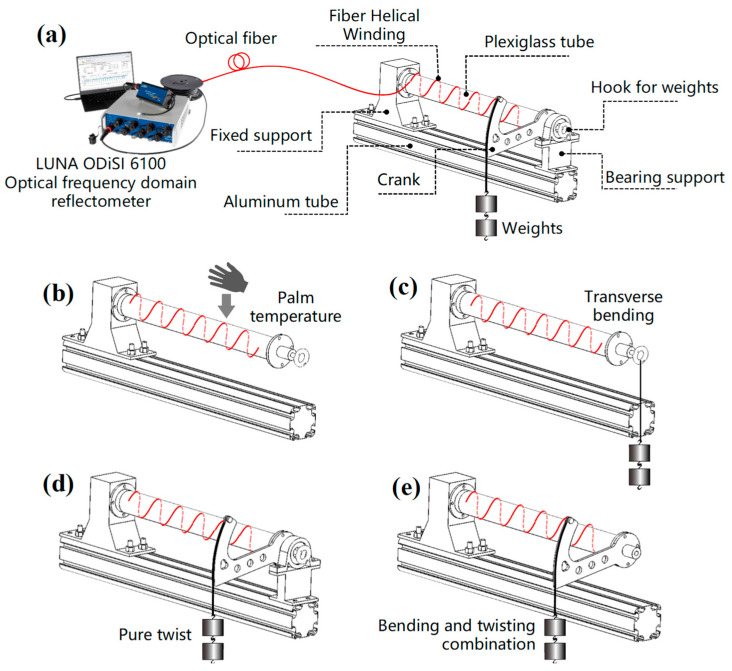
Schematic diagram of the experiment setup and four typical load application: (**a**) experimental device; (**b**) palm-shaped temperature load; (**c**) transverse bending load; (**d**) pure torsion load; (**e**) bending and torsion combination load.

**Figure 9:**
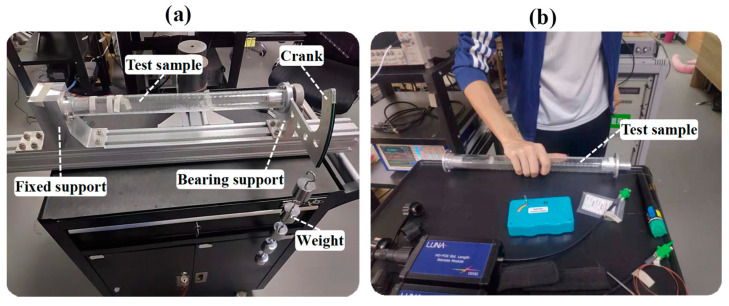
Physical picture of the experiment setup: (**a**) experimental device; (**b**) temperature load application.

As shown in Figure 8a, we used an organic glass cylinder with an outer diameter of 36 mm, a length of 300 mm, and a wall thickness of 4 mm. A 3.5 m-long polyimide-coated single-mode optical fiber was helically wound with a pitch of 10 mm and adhered to the cylinder surface. One end of the tube was fixed on a bracket, and the other end could achieve three working conditions of transverse bending deformation, pure torsion deformation, and bending-torsion combination deformation through different supports and load situations. Figure 9 shows the physical picture of the experimental device. In addition, the sample in the free state can measure the complex temperature field. These load scenarios simulate different working conditions that may be encountered in actual engineering applications, making the experimental results more representative and generalizable.

## 4. Experimental Results

The spatial resolution of ODiSI equipment acquisition is set to 0.65 mm, and a total of about 5400 measurement points are obtained on the 3.5 m optical fiber. The surface of a cylinder with a diameter of 36 mm and a length of 300 mm is approximately 340 cm^2^. On average, there are 15.9 measuring points per square, which provides a large amount of data for reconstructing the temperature and strain fields.

### 4.1. Palm-Shaped Temperature Field Reconstruction

**Figure 10:**
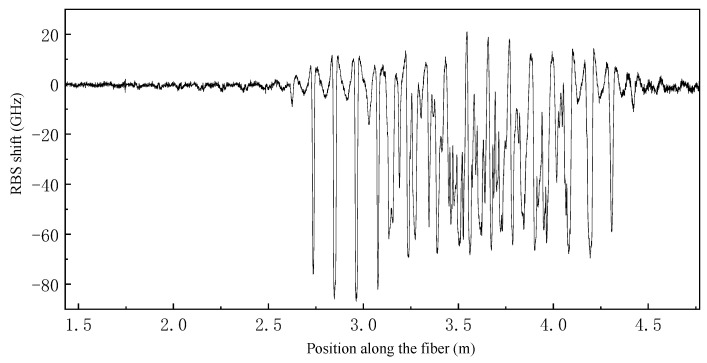
Test results of palm temperature in fiber arc length coordinates.

Temperature field reconstruction experimentation was conducted on the sample in the stress-free state. First, the sample was held in the hand, as shown in Figure 8b and Figure 9b, and kept for about 10 s to fully transfer the palm temperature to the surface of the cylinder. Then, the fiber data were extracted at this moment, as shown in Figure 10. The fiber RBS shift signal is given in the form of arc length and is difficult to recognize directly, so it must be reconstructed using the method mentioned above.

**Figure 11:**
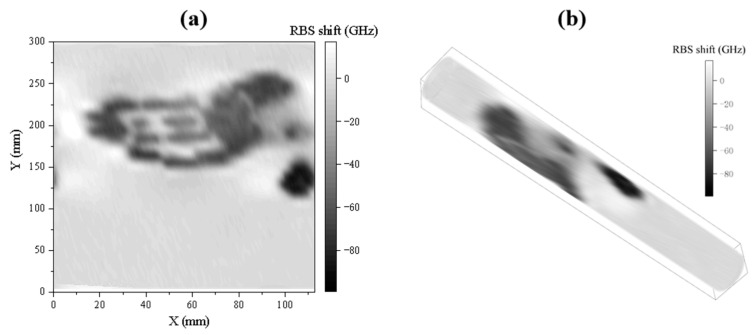
Palm temperature distribution reconstruction: (**a**) temperature distribution of cylindrical side expansion; (**b**) cylindrical surface temperature distribution.

The reconstructed two-dimensional figure is shown in Figure 11, and the outline of the palm is clear and complete. At the joints and the center of the palm, the temperature is lower due to insufficient contact and appears light in color; in other parts, it presents corresponding grayscale based on the palm temperature and contact degree. It is worth noting that because the fiber is attached to the organic glass tube, the signal it perceives includes the thermal deformation of the organic glass and the thermal-optical effect of the fiber. Therefore, the obtained signal is related to temperature and can correspond to specific temperature values after calibration. These results show that the proposed reconstruction method can effectively reconstruct the temperature distribution on the cylindrical surface, providing a reliable temperature field monitoring means for practical applications.

### 4.2. Transverse Bending Strain Field Reconstruction

The tower tube of the fan is subjected to strong wind, and the pile foundation is subjected to ocean currents and waves, which can be abstracted as cylindrical shells subjected to transverse bending. In this experiment, we studied the impact of transverse bending force on the strain field. As shown in Figure 8c, the bearing support of the mechanical loading device was removed, and a 1 kg weight was directly hung on the hook at the end of the tube. This setup allowed the tube to bend under the effect of the transverse force. Then, we measured the resulting strain field and performed reconstruction.

**Figure 12:**
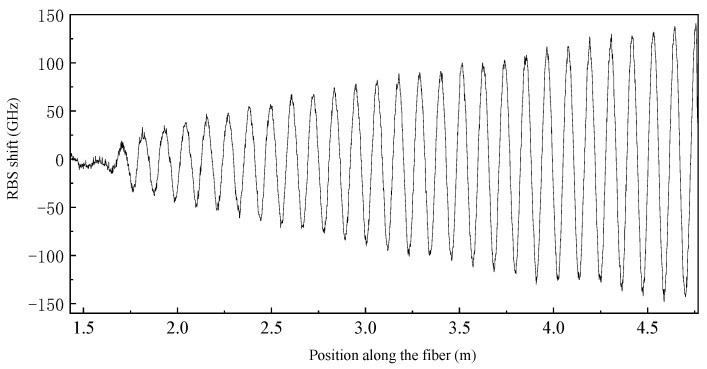
Transverse bending test results under fiber arc length coordinates.

As shown in Figure 12, along the direction of the fiber length, the RBS shift signal shows a stable sinusoidal periodic fluctuation, and the amplitude linearly amplifies. The two envelope lines are approximately symmetrically distributed around RBS shift = 0. The results showed that transverse bending force had a significant impact on the strain field.

**Figure 13:**
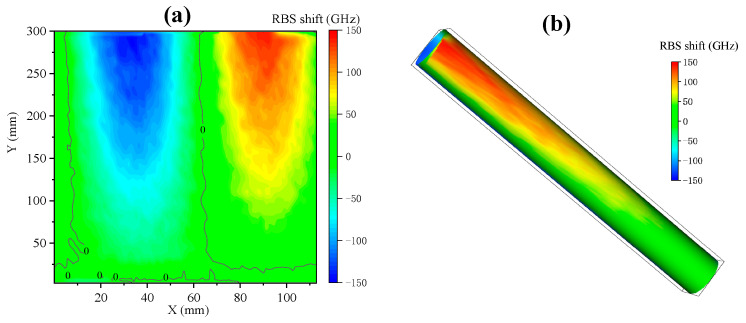
Transverse bending strain field reconstruction: (**a**) expanded strain distribution on the side of the cylinder; (**b**) cylindrical surface strain distribution.

The two-dimensional reconstructed signal is shown in Figure 13. Under the action of transverse force, the cylindrical surface of the cantilever beam presents a symmetrical stress distribution, and the tensile stress and compressive stress on both sides of the neutral plane are equal. In Figure 13a, with the 0 contour line as the boundary, red and blue areas of the same shape are presented on both sides. The closer to the fixed end, the larger the bending moment, and the color gradually deepens. Figure 13b shows the cylindrical surface strain distribution after reconstruction. The values given by the test data are the RBS shifts caused by the strain in the direction of the spiral line tangent. Through simple calculations, the strain state in each direction can be obtained. These results show that the proposed reconstruction method can effectively reconstruct the strain field under transverse bending force, providing a reliable means of strain monitoring for practical applications.

### 4.3. Pure Torsion Strain Field Reconstruction

When the direction of the impeller of the wind turbine is not consistent with the wind direction, the tower will be subjected to strong torsion, which can be abstracted as the torque acting on the end of the cylinder. In this experiment, we studied the strain field reconstruction under pure torsion.

**Figure 14:**
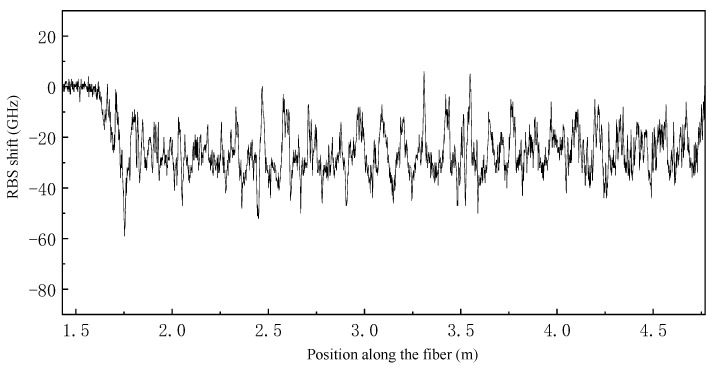
Pure torsion test results under fiber arc length coordinates.

As shown in Figure 8d, we used the bearing bracket to eliminate the bending moment caused by the weights, making the sample close to a pure torsion state. By applying a 1 kg weight on the crank, we collected the fiber signal, as shown in Figure 14. Due to the precision of the bracket processing, there is still some bending moment remaining in the sample under the ideal pure torsion state, so there is still sinusoidal fluctuation in the local signal. However, the overall signal is negative, indicating that torsion is the dominant factor. In the main test section, the mean value of RBS shift is about −26.7 GHz.

**Figure 15:**
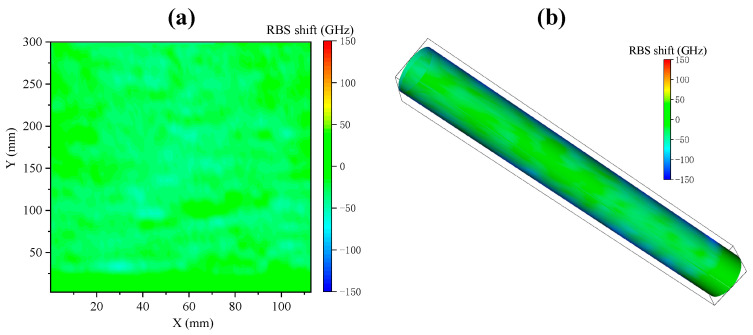
Pure torsion strain field reconstruction: (**a**) expanded strain distribution on the side of the cylinder; (**b**) cylindrical surface strain distribution.

The reconstructed signal is shown in Figure 15. Since the torque is equal at all points of the cylinder, the entire surface is mainly subjected to circumferential shear strain, and the main strain direction is at a 45° angle with the axial direction. The angle between the tangent direction of the fiber and the main strain direction is fixed, so the tensile strain at each point of the fiber is constant. Therefore, the overall reconstruction appears bluish-green, without obvious regional color differences, indicating that the strain along the fiber tangent direction on the surface of the cylinder is relatively uniform. These results verify the effectiveness of the proposed reconstruction method in the reconstruction of the pure torsion strain field, providing strong support for subsequent applications in more complex scenarios.

### 4.4. Combined Bending and Torsion Strain Field Reconstruction

**Figure 16:**
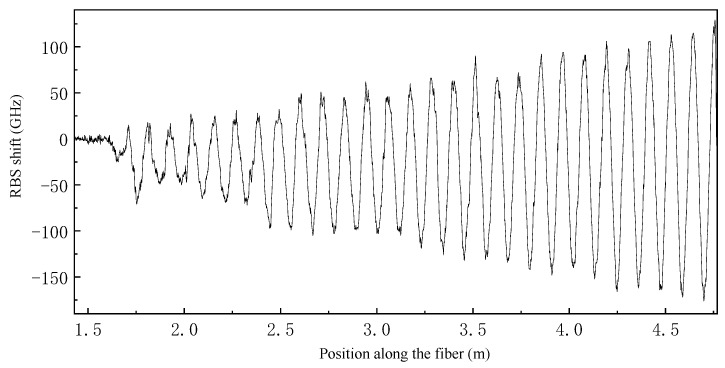
Combined bending and torsion deformation test results under fiber arc length coordinates.

In engineering practice, bending and torsional loads often appear at the same time, and it is of more engineering value to consider this situation. As shown in Figure 8e, by removing the bearing support and hanging a 1 kg weight on the crank, the purpose of superimposing bending and torsional loads on the plexiglass tube was achieved. Different from the situation of single transverse bending, after bending and torsion are superimposed, the curve as a whole moves downwards, and the amount of displacement (−27 GHz) is roughly equal to the signal under single torsion (−26.7 GHz). After superimposition, the signal is centered on RBS shift =−27 GHz, showing sinusoidal fluctuations, and the amplitude gradually increases, as shown in Figure 16.

**Figure 17:**
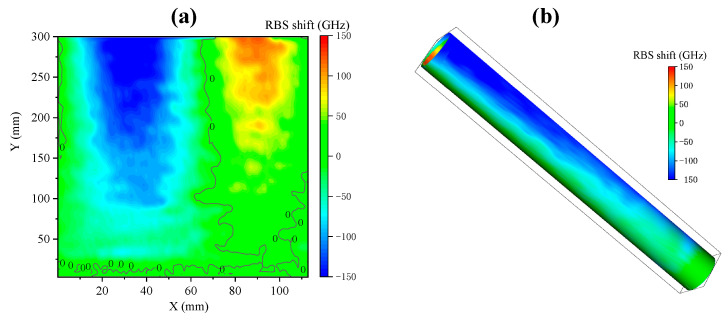
Combined bending and torsion deformation strain field reconstruction: (**a**) expanded strain distribution on the side of the cylinder; (**b**) cylindrical surface strain distribution.

In the cloud map after the reconstruction of the bending and torsion combined deformation, the difference in the area of the tension and compression region is significant, and the blue area is significantly larger than the red area. In the case of bending alone, the areas of tension and compression are equal (Figure 13), while single torsion will generate a uniform tension signal (Figure 15). Therefore, after superimposition, the tension area will be larger than the compression area, and there will be a shift in the neutral plane (Figure 17).

## 5. Conclusions

This study has delved into the application of distributed fiber optic sensing technology for the three-dimensional curved surface strain measurement, evaluating its potential for strain field monitoring of wind turbines. The results demonstrate that our method of implementing a spiral configuration of optical fibers allows us to obtain 15.9 measurement points per square centimeter on the cylindrical surface. This high-density measurement approach facilitates the effective reconstruction of temperature and strain fields. For the diverse tests conducted—including transverse force bending, pure torsion, combined bending and torsion loads, and palm temperature loads—the reconstructed strain and temperature distribution graphics all align with the anticipated effects of actual loads.

Although this approach has only been tested on small-scale lab structures at present, it lays a strong groundwork for future research in real wind turbine applications. However, we anticipate certain challenges, including: rough surfaces affecting the precision of fiber optic installation; increased fiber lengths potentially augmenting the risk of signal loss; larger-scale data requiring enhanced computer performance; and ongoing efforts to identify durable, reliable methods for fiber optic installation on wind turbine structures.

## Data Availability

Data is contained within the article.
